# Floral Resources for *Trissolcus japonicus*, a Parasitoid of *Halyomorpha halys*

**DOI:** 10.3390/insects11070413

**Published:** 2020-07-03

**Authors:** Hanna R. McIntosh, Victoria P. Skillman, Gracie Galindo, Jana C. Lee

**Affiliations:** 1 Department of Entomology, University of Wisconsin-Madison, Madison, WI 53706, USA; 2 Horticulture Crops Research Unit, USDA-ARS, Corvallis, OR 97331, USA; gracie.galindo@usda.gov (G.G.); jana.lee@usda.gov (J.C.L.); 3 Hermiston Agricultural Research and Extension Center, Oregon State University, Hermiston, OR 97838, USA; skillmav@oregonstate.edu

**Keywords:** biological control, biocontrol, brown marmorated stink bug, conservation, flowers, survival, longevity, nectar subsidy, samurai wasp

## Abstract

The egg parasitoid *Trissolcus japonicus* is the main candidate for classical biocontrol of the invasive agricultural pest *Halyomorpha halys*. The efficacy of classical biocontrol depends on the parasitoid’s survival and conservation in the agroecosystem. Most parasitoid species rely on floral nectar as a food source, thus identifying nectar sources for *T. japonicus* is critical. We evaluated the impact of eight flowering plant species on *T. japonicus* survival in the lab by exposing unfed wasps to flowers inside vials. We also measured the wasps’ nutrient levels to confirm feeding and energy storage using anthrone and vanillin assays adapted for *T. japonicus*. Buckwheat, cilantro, and dill provided the best nectar sources for *T. japonicus* by improving median survival by 15, 3.5, and 17.5 days compared to water. These three nectar sources increased wasps’ sugar levels, and cilantro and dill also increased glycogen levels. Sweet alyssum, marigold, crimson clover, yellow mustard, and phacelia did not improve wasp survival or nutrient reserves. Further research is needed to determine if these flowers maintain their benefits in the field and whether they will increase the parasitism rate of *H. halys*.

## 1. Introduction

The brown marmorated stink bug, *Halyomorpha halys* (Stål), is a polyphagous agricultural pest that has spread from its native range in Asia to the United States [1], Canada [2], Europe [3], and Chile [4] in the last 25 years. *Halyomorpha halys* feeds on cultivated fruits, vegetables, row crops, ornamentals, and wild host plants [5]. Due to its wide host range, dispersal capacity, and voracious feeding by nymphs and adults, *H. halys* causes substantial economic damage [5,6]. Infestations are largely controlled by insecticides, which is costly to growers, has limited efficacy, is not compatible with integrated pest management strategies [7], and risks insecticide resistance and secondary pest outbreaks. Management is especially difficult in organic production since fewer effective insecticides are approved [8]. Thus, recent research aims to develop alternative management practices, including classical biological control.

The samurai wasp, *Trissolcus japonicus* (Ashmead), is the main candidate for classical biocontrol of *H. halys*. This egg parasitoid can kill up to 70% of *H. halys* eggs in the insects’ native range [9]. *Trissolcus japonicus* has been studied in quarantine as a potential biocontrol agent for *H. halys* since 2017 [6], but adventive populations genetically distinct from those in quarantine were found in Maryland [10] and Washington State [11] in 2014 and 2015. Since then, *T. japonicus* has been detected in Virginia, West Virginia, Delaware, New Jersey, New York, the District of Columbia [5], Oregon [12], Ohio, Pennsylvania [5], and Michigan [13]. While *T. japonicus* continues to spread to new areas in the United States, its parasitism rate remains low. In New Jersey, the parasitism rate was 2.2% in peach and 0% in apple [14]. More research is needed to improve biocontrol, including strategies for rearing, release, augmentation, and conservation. The efficacy of *T. japonicus* as a biocontrol agent depends on its survival and conservation in the agroecosystem.

Identifying nectar sources for *T. japonicus* is critical since most parasitoid species feed on floral nectar [15]. Floral nectar sources have also shown to improve parasitoid longevity or fecundity in the field [16,17], thus biocontrol may be improved by nectar subsidies [18]. However, simplified agroecosystems may not provide the right plant species or may lack temporal continuity of flowering plants. Further, not all flowers are sufficient nectar sources for parasitoids. Flowers with long or complex corollas inhibit foraging by small wasps with short mouthparts [19,20,21,22]. Furthermore, not all nectar has optimal nutritional characteristics for parasitoids, which usually depend on specific amino acid compositions and sucrose to hexose ratios [23]. Thus, there is a need to identify plants that provide suitable nectar sources for the *T. japonicus* to help improve survival and conservation in the agroecosystem.

This study tested eight flowering plants as potential nectar sources for *T. japonicus*: sweet alyssum (*Lobularia maritima* (L.) Desv.), marigold (*Tagetes patula* L.), buckwheat (*Fagopyrum esculentum* Moench), cilantro (*Coriandrum sativum* L.), crimson clover (*Trifolium incarnatum* L.), dill (*Anethum graveolens* L.), yellow mustard (*Guillenia flavescens* (Hook.) Greene), and phacelia (*Phacelia tanacetifolia* Benth.). We evaluated the impact of each of these eight plants on *T. japonicus* survival in the lab. Honey water was used as a positive control, since *T. japonicus* can live over 40 d on honey water in the laboratory [24]. A review of floral resources for parasitoids found that many of these plants or closely-related species are attractive to parasitoids and improve longevity [15]. Further, buckwheat, marigold, cilantro, and phacelia were shown to improve longevity of *Trissolcus basalis*, another important parasitoid of stinkbugs [25]. We were also interested in marigold due to its allelopathic suppression of parasitic nematodes [26] and ability to improve the parasitism rate of *Aphidius platensis* Brethes [27]. To confirm that wasps fed on flowers and gained energy reserves, lipid, glycogen, and sugar levels were measured in wasps following feeding [28].

## 2. Materials and Methods

### 2.1. Insect Colonies

All colonies were maintained at the USDA ARS Horticultural Crops Research Unit in Corvallis, OR. The *H. halys* colony was started and maintained from wild-collected bugs. Adults and nymphs were collected via beat sheet from May to November at multiple sites across the Willamette Valley, mostly from cultivated holly plants (*Ilex aquifolium).* When wild bugs were in winter diapause, adults were collected from aggregations in local residences. The colony was replenished with new adults throughout the year, so that all individuals were wild-collected and none were reared from eggs laid in the lab colony. Bugs were contained in 29.5 × 29.5 × 30.5 cm plastic mesh cages (Bug Dorm, BioQuip, Rancho Dominguez, CA, USA) inside a growth chamber (21–22 °C, 16:8 L:D, 60–70% RH). Each cage included 20–80 adults. Each cage contained carrots, raw unsalted peanuts and/or sunflower seeds, jellybeans, and a water wick.

The *T. japonicus* colony was established in 2017 from field populations in Portland, Oregon, collected from sentinel and wild egg masses. Wasps were contained in ~473 mL plastic-lined paper soup cups with plastic lids (Huhtamaki Inc., Espoo, Finland). Four colony cups were stored inside 29.5 × 29.5 × 30.5 cm Bug Dorm cages in the lab (~21 °C, natural day length conditions, ~60% RH). A small section of filter paper soaked in 50% honey/water solution was provided each weekday, along with 4–8 *H. halys* egg masses per week. We used fresh egg masses when available and supplemented with frozen egg masses when necessary [29].

### 2.2. Survival/Longevity

We tested the impact of 8 flowers on *T. japonicus* survival in a series of trials in the lab (Table 1), which were conducted based on flower availability. Treatments in trial 1 included sweet alyssum, Nema-gone marigold (Burpee, Warminster, PA, USA), honey (positive control), and water (negative control). Treatments in trial 2 included buckwheat, honey, and water. Treatments in trial 3 included cilantro, crimson clover, dill, mustard, phacelia, honey, and water. All flowers were grown from seed in the Professional Growing Mix (Sun Gro^®^ Horticulture, Agawam, MA, USA) in a greenhouse at the USDA ARS Horticulture Research Unit in Corvallis, OR, USA.

Treatment vials were set up on three or more dates with emerging wasps divided between treatments (see Table 1 for vial and wasp numbers and dates). Treatments were set up in 9 × 3 cm (diameter) plastic vials with cotton plugs and 2 mL microcentrifuge tube ‘vases’ glued inside. Vases were filled with water, and flowers were inserted wrapped in cotton to prevent wasps from drowning. Each vase contained one stem of a single flower treatment. Water control vials included only an empty vase of water plugged with cotton. Honey control vials did not contain a vase and had a small piece of filter paper dipped into a 50:50 honey:water solution stuck to the inside of the vial.

After flower set-up, 3–8 newly emerged (<1 d old), unfed *T. japonicus* were introduced into each vial. All vials included adult females and males, but the number of each sex varied by availability in the colony. To promote normal oviposition behavior, all vials contained one *H. halys* egg mass (<3 d old at harvest, stored at −80 °C) affixed to the cotton plug with double-sided tape; egg masses were changed weekly. Flowers were changed and vases were refilled every Monday, Wednesday, and Friday. Honey was changed every weekday.

After assembly, wasps were monitored every weekday for mortality. Wasps that were missing, stuck in honey or water, or had escaped during flower changing were marked as such and censored in the data set. To account for unknown day of death on weekends, wasps recorded as dead on Mondays were marked and 1.5 was subtracted from their day of death. Trials 1 and 2 were ended after 30 days or earlier if all wasps died. Wasps surviving to 30 days were marked as censored. For trial 3, we wanted to assess longevity more comprehensively, thus trials ended after all wasps died.

### 2.3. Nutrient Levels

Wasps exposed to the 8 flower treatments were measured for nutrient levels to confirm feeding and energy storage. Only female wasps were tested since their feeding would extend their oviposition period. Trial 4 combined treatments from survival trials 1 and 2 (sweet alyssum, marigold, buckwheat, honey, water). Trial 5 used the same treatments as longevity trial 3 (cilantro, crimson clover, dill, mustard, phacelia, honey, water). Vials were set up as described for the survival studies with newly emerged wasps, except flowers were not changed and egg masses were not provided. Wasps were removed from vials after 48 h and frozen at −80 °C.

Lipid, glycogen, and total sugar levels were measured following anthrone and vanillin assays [30,31] adapted for *T. japonicus* [28]. Protocols were adjusted to have 250 µL reaction volumes due to the small size of *T. japonicus*. Individual wasps were crushed in 13 µL sodium sulfate in a 1.5 mL microcentrifuge tube with a pestle. Next, 113 µL of chloroform:methanol (1:2) was added, vortexed, and centrifuged for 2 min at 13,000 rpm. The supernatant was poured into a glass tube, and the glycogen precipitate was reacted with 240 µL of anthrone reagent by vortexing and heating at 90 °C for 3 min. After cooling on ice, the glycogen samples were pipetted into 96-well plates (Costar 3590, Corning Inc., Corning, NY, USA) and read at 630 nm on a spetrophotometer (BioTek ELX, BioTek Instruments, Winooski, VT, USA). The supernatant was divided in half into two glass tubes. One tube was evaporated at 90 °C for 1–2 min until 10 µL of solution remained and was reacted with 240 µL anthrone at 90 °C for 3 min. Cooled sugar samples were read on a spectrophotometer at 630 nm. The other supernatant tube was evaporated at 90 °C for 3–4 min until nearly dry. Then, 10 µL of sulfuric acid was added and heated at 90 °C for 30 s. Once cooled, 240 µL of vanillin reagent was added and reacted for 20 min at room temperature. Lipid samples were read on a spectrophotometer at 490 nm.

Standard calibrations were made for each reagent to later convert absorbance readings from wasps. The lipid calibration included three replicates of 1, 5, 10, 15, and 20 µg (=1 µL) of lipid (50 mg canola oil in 50 mL chloroform) and control. The same procedures were followed to evaporate the solution, heated with sulfuric acid, reacted with vanillin in a 250 µL reaction volume, and read on a spectrophotometer. The sugar (50 mg sucrose in 50 mL of 25% ethanol) and glycogen (50 mg beef liver (Fisher Scientific, Waltham, MA, USA) in 50 mL purified water) calibration included three replicates of 1, 5, 10, 15, and 20 µg and control. The 20 µg (µL) sample was briefly heated at 90 °C to bring the volume down. Anthrone reagent was added for a final volume of 250 µL, heated at 90 °C for 5 min, and read on a spectrophotometer.

### 2.4. Data Analysis

For survival studies, Kaplan–Meier survival curves were generated and tested by log-rank analyses using PROC LIFETEST [32]. Data were analyzed separately for each trial and each sex. Combining both sexes may skew results since there were variable ratios of sexes per treatment and we observed females to live longer than males. Post-hoc tests compared each pair by log-rank analysis with an adjusted Sidak *p*-value.

To convert absorbance readings from wasps to nutrient values, the relationships between nutrient mass (µg, *x*-axis) and absorbance (*y*-axis) from standard calibrations were fitted to linear models with the intercept at 0 using JMP 15.0. For lipids, the slope calculated was 0.0327 (*p* < 0.0001, r^2^ = 0.901) and 0.0233 (*p* < 0.0001, r^2^ = 0.948) in 2018 and 2019, respectively. For glycogen, the slope was 0.051 (*p* < 0.0001, r^2^ = 0.741) and 0.107 (<0.0001, r^2^ = 0.823) in 2018 and 2019, respectively. For sugars, the slope was 0.194 (*p* < 0.0001, r^2^ = 0.951) and 0.191 (*p* < 0.0001, r^2^ = 0.951) in 2018 and 2019, respectively. Absorbance readings from each wasp were divided by the slope from the calibration line to estimate nutrient content. Since the supernatant was divided in half, sugar and lipid readings were multiplied by two. Nutrient levels of wasps were analyzed by generalized linear mixed models with treatment as a fixed effect and vial as a random effect using a lognormal distribution in PROC GLIMMIX [32]. Dunnett tests were used to compare treatments to the water control. Separate analyses were done by nutrient (lipid, glycogen, or sugar), sex, and trial. Tukey post-hoc tests were used for multiple pairwise comparisons.

## 3. Results

All raw data is included in Table S1. 

### 3.1. Survival/Longevity

**Figure A1:**
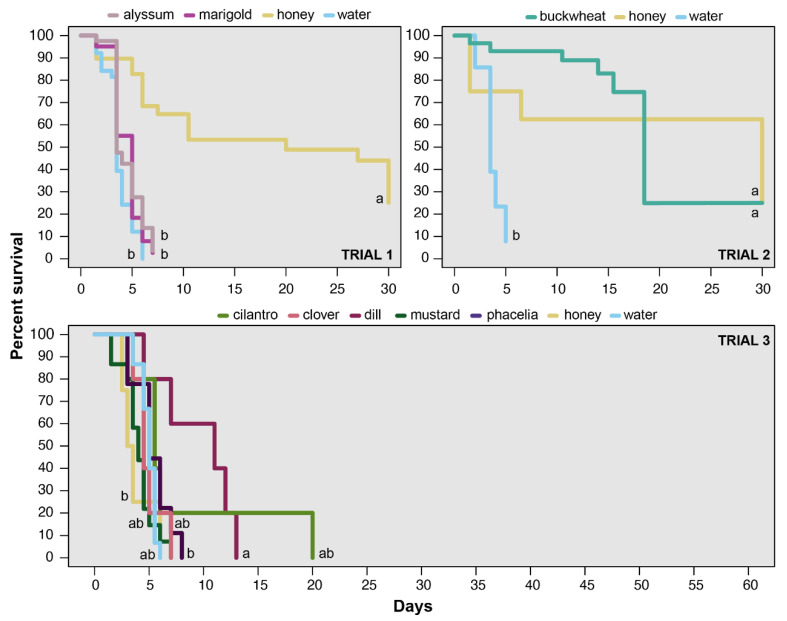
Survival curves of male *Trissolcus japonicus* fed flower treatments, honey, or water inside vials in the lab. Per results of log-rank analysis and post-hoc tests, lines with different letters are significantly different from each other.

Results for females are presented here. Results for males are given in the Appendix A, since the results are similar but had low replication due to the 80:20 female:male sex ratio of the colony (Table A1, Figure A1). Moreover, the longevity of males is less critical since males patrol egg masses waiting to mate with emerging females.

**Figure 1:**
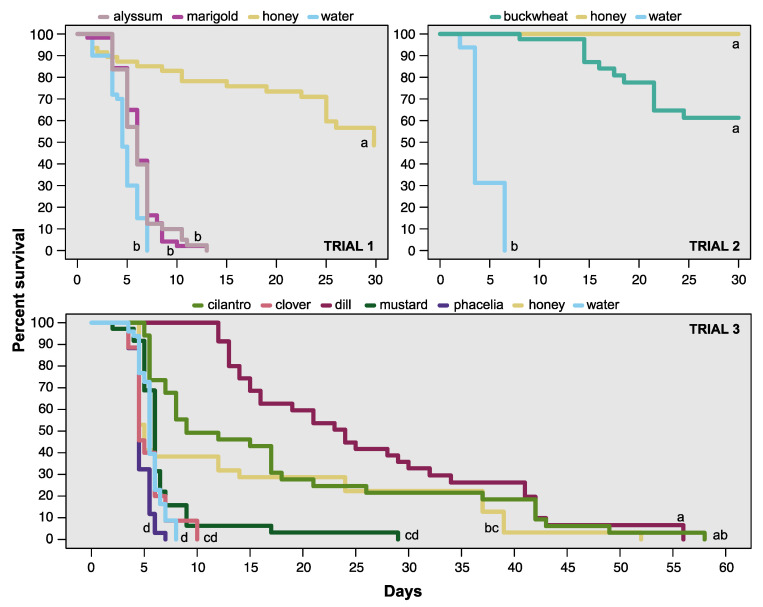
Survival curves of female *Trissolcus japonicus* fed with flower treatments, honey, or water inside vials in the lab. Per results of log-rank analysis and post-hoc tests, lines with different letters are significantly different from each other.

In trial 1, treatment impacted female *T. japonicus* survival (χ^2^ = 110.98, df = 3, *p* < 0.0001; Figure 1). Wasps fed with honey survived significantly longer than wasps fed with water, as expected. Wasps fed with alyssum or marigold did not survive longer than the water control. The median survival for females, when 50% of wasps were alive, was 4.5 days when fed with water, 6 days when fed with marigold or alyssum, and 28 days when fed with honey.

In trial 2, the treatment impacted female *T. japonicus* survival (χ^2^ = 101.99, df = 2, *p* < 0.0001; Figure 1), and wasps fed with buckwheat survived significantly longer than wasps fed only with water. Survival on buckwheat was equivalent to survival on honey. The median survival for females was 3.5 days when fed with water, and more than 50% of wasps were still alive at 30 days when fed with buckwheat or honey.

In trial 3, the treatment again impacted female survival (χ^2^ = 153.11, df = 6, *p* < 0.0001; Figure 1), and wasps survived longest on cilantro, dill, and honey. Survival on crimson clover, mustard, and phacelia was not significantly different than the water control. The median survival for females was 5.5 days when fed with water, 4.5 days when fed with crimson clover or phacelia, 6 days when fed with mustard, 9 days when fed with cilantro, 24 days when fed with dill, and 5 days when fed with honey.

### 3.2. Nutrient Levels

**Figure 2:**
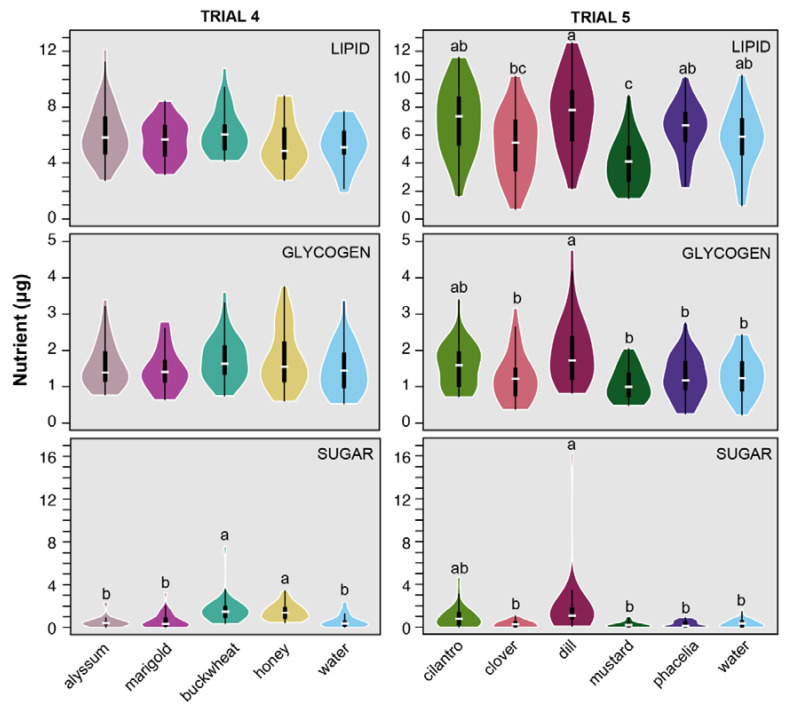
Kernel density plots of nutrient levels of female *Trissolcus japonicus* fed with flower treatments, honey, or water for 48 h inside vials in the lab. Internal boxplots show the median, interquartile range, maximum, and minimum. Treatments with different letters are significantly different based on Tukey post-hoc tests.

In trial 4, there was no significant difference in lipid (df = 4, 111; F = 1.34; *p* = 0.26) or glycogen (df = 4, 111; F = 0.65; *p* = 0.63) levels between treatments (Figure 2). Sugar levels were equivalent for wasps fed with buckwheat and honey, and both were significantly higher than the water control (df = 4, 111; F = 7.65; *p* < 0.001).

In trial 5, the lipid levels differed by treatment (df = 5, 167; F = 8.07; *p* < 0.001; Figure 2) and were highest for wasps fed with dill, but not significantly different than wasps fed with water, cilantro, or phacelia. Lipid levels for wasps fed with mustard were significantly lower than water controls. The lipid levels for wasps fed with crimson clover were intermediate between mustard and water. Glycogen (df = 5, 167; F = 4.14; *p* = 0.0014) and sugar (df = 5, 167; F = 7.99; *p* < 0.001) levels differed by treatment and were substantially higher for wasps fed with dill. Wasps fed with water, phacelia, mustard, and clover had equivalent glycogen and sugar levels. Wasps fed with cilantro were intermediate between water and dill.

## 4. Discussion

Of the flowers tested, buckwheat, cilantro, and dill provided the best nectar source for *T. japonicus*, improving the median survival by 15, 3.5, and 18.5 days compared to water, respectively. Wasps survived a maximum of 58 and 56 days on cilantro and dill, respectively. The maximal survival on buckwheat was not recorded since trial 2 ended after 30 days, but 61% of female wasps were still alive at 30 days. Feeding on buckwheat was equivalent to feeding on honey, but the results for honey were less consistent in trial 3 when wasps fed with honey did not survive as long as honey-fed wasps in the previous trials. The honey/water solution used in trial 3 may have been somehow compromised. We are confident that the other treatments in trial 3 were not compromised, since those wasps survived as expected. Wasps in trial 3 survived up to six to eight days on water as in trials 1 and 2, and some wasps with cilantro or dill survived past 30 days as wasps given buckwheat or honey in trials 1 and 2.

Feeding on buckwheat, cilantro, and dill increased the wasps’ sugar levels, which provides further confirmation of benefits of nectar feeding. Glycogen levels were increased by cilantro and dill compared to water, but not buckwheat during a two-day feeding period. This suggests that ingested cilantro and dill nectar was converted to glycogen for longer-term storage. Increased glycogen reserves in *T. japonicus* are likely beneficial since glycogen fuels flight and is important for overwintering in other insect species [33,34].

Nectar from buckwheat, dill, and cilantro have similarly extended longevity in other parasitoid species. When averaged over multiple studies, parasitoids fed with buckwheat showed one of the highest increases in longevity compared to other flowers, wasps fed with dill showed a moderate increase in longevity, and wasps fed with cilantro showed a small increase in longevity [15]. As in our study, wasps fed with clover (*Trifolium pretense* and *Trifolium repens*), mustard, and phacelia showed a negligible increase in longevity. Alyssum did not improve longevity for *T. basalis* [25]. Contrary to our study, Russell [15] reported marigold as providing a very high increase in longevity and also showed alyssum providing a moderate longevity increase in other wasp species.

Floral architecture is likely to explain the relative success of certain floral resources for *T. japonicus*. Corolla length and corolla opening diameter are determining factors for whether small parasitoids can access nectar [22,35]. We observed that *T. japonicus* were too wide and could not enter the narrow flowers of crimson clover, though they may access these nectaries in the field if bees have opened up the flowers. Flowers with shallow corollas are best for small wasps [36], which may have difficulty crawling into longer flowers. Further, across multiple studies, plants that conferred the greatest longevity benefit to parasitoids all had exposed nectaries [15]. Indeed, buckwheat, dill, and cilantro all have short corollas and exposed nectaries.

While our study has identified three promising nectar sources for *T. japonicus*, additional research is needed to determine if these flowers maintain their ability to improve wasp survival in the field, and whether they will increase the parasitism rate of *H. halys*. Flowers that improve parasitoid longevity do not always increase the parasitism rate [18]. For example, buckwheat floral borders extended the longevity of *Diadegma insulare* (Cresson) (Hymenoptera: Ichnuemonidae) females and males foraging in the field, but only marginally increased parasitism rates [16]. However, buckwheat improved the fecundity of *Trissolcus basalis,* and buckwheat row margins improved the parasitism of host eggs [37,38]. Floral provisioning in agroecosystems provides nutrients to many important predators and parasitoids and is generally recommended for the conservation of biological control [39] and thus merits further testing for *H. halys* management.

## 5. Conclusions

Sustainable management of *H. halys* in organic and conventional agriculture requires the development of alternative management practices. Classical biocontrol via the release of *T. japonicus* is promising but may be constrained by the wasp’s survival and conservation in the agroecosystem. Buckwheat, cilantro, and dill are promising nectar subsidies for *T. japonicus* since they improve wasp survival and increase nutrient reserves. To determine if these flowers will help facilitate successful biocontrol, they need to be tested in field trials to determine if they improve the survival and longevity of *T. japonicus* and increase the parasitism of *H. halys* eggs.

## Figures and Tables

**Table 1:** Summary of trials. Newly emerged, unfed wasps were placed in vials with each treatment and evaluated for 30 d (trials 1 and 2) or until all wasps were dead (trial 3). For nutrient trials, wasps were fed for 48 h and nutrients were measured using anthrone and vanillin assays adapted for *Trissolcus japonicus*.

Trial	Measurement	Sex	Treatment (Number of Wasps, Number of Vials)	Dates
1	survival (30 d)	f	alyssum (51, 15), marigold (58, 16), honey (47, 13), water (50, 14)	May–August 2017
		m	alyssum (41, 15), marigold (41, 16), honey (29, 13), water (36, 14)	
2	survival (30 d)	f	buckwheat (49, 13), honey (15, 3), water (16, 3)	September 2017–March 2018
		m	buckwheat (29, 13), honey (8, 3), water (14, 3)	
3	survival (until dead)	f	cilantro (35, 5), crimson clover (35, 5), dill (35, 5), mustard (36, 7), phacelia (35, 5), honey (35, 5), water (99, 15)	June–September 2019
		m	cilantro (5, 5), crimson clover (5, 5), dill (5, 5), mustard (11, 7), phacelia (5, 5), honey (15, 5), water (15, 15)	
4	nutrient levels (2 d)	f	alyssum (30, 9), marigold (30, 7), buckwheat (30, 7), honey (30, 9), water (30, 9)	6–26 June 2018
5	nutrient levels (2 d)	f	cilantro (30, 4), crimson clover (30, 4), dill (30, 4), mustard (30, 5), phacelia (30, 4), water (50, 9)	June–July 2019

## Supplementary Materials

The following are available online at https://www.mdpi.com/2075-4450/11/7/413/s1, Table S1: *Trissolcus japonicus* floral resource data.

## Appendix A

**Table A1:** Statistical results for male *Trissolcus japonicus* in trials 1–3 testing survival. Treatment differences were assessed using log-rank analysis.

Trial	χ^2^	df	*p* < χ^2^
1	52.06	3	<0.001
2	28.6	2	<0.001
3	18.35	6	0.0054
